# Oligomer-prone E57K-mutant alpha-synuclein exacerbates integration deficit of adult hippocampal newborn neurons in transgenic mice

**DOI:** 10.1007/s00429-017-1561-5

**Published:** 2017-11-09

**Authors:** Martin Regensburger, Sebastian R. Schreglmann, Svenja Stoll, Edward Rockenstein, Sandra Loskarn, Wei Xiang, Eliezer Masliah, Beate Winner

**Affiliations:** 1 Department of Stem Cell Biology, Friedrich-Alexander-Universität Erlangen-Nürnberg (FAU), Erlangen, Germany; 2 IZKF Junior Research Group III, and BMBF Research Group Neuroscience, FAU, Erlangen, Germany; 3 Department of Neurology, FAU, Erlangen, Germany; 4 School of Medicine, University of Regensburg, Regensburg, Germany; 5 Department of Neurosciences, University of California, San Diego, La Jolla, CA USA; 6 Institute of Biochemistry, FAU, Erlangen, Germany; 7 Present Address: Sobell Department of Motor Neuroscience and Movement Disorders, Institute of Neurology, UCL, London, UK

**Keywords:** Alpha-synuclein, Adult neurogenesis, Hippocampus, Oligomers

## Abstract

In the adult mammalian hippocampus, new neurons are constantly added to the dentate gyrus. Adult neurogenesis is impaired in several neurodegenerative mouse models including α-synuclein (a-syn) transgenic mice. Among different a-syn species, a-syn oligomers were reported to be the most toxic species for neurons. Here, we studied the impact of wild-type vs. oligomer-prone a-syn on neurogenesis. We compared the wild-type a-syn transgenic mouse model (Thy1-WTS) to its equivalent transgenic for oligomer-prone E57K-mutant a-syn (Thy1-E57K). Transgenic a-syn was highly expressed within the hippocampus of both models, but was not present within adult neural stem cells and neuroblasts. Proliferation and survival of newly generated neurons were unchanged in both transgenic models. Thy1-WTS showed a minor integration deficit regarding mushroom spine density of newborn neurons, whereas Thy1-E57K exhibited a severe reduction of all spines. We conclude that cell-extrinsic a-syn impairs mushroom spine formation of adult newborn neurons and that oligomer-prone a-syn exacerbates this integration deficit. Moreover, our data suggest that a-syn reduces the survival of newborn neurons by a cell-intrinsic mechanism during the early neuroblast development. The finding of increased spine pathology in Thy1-E57K is a new pathogenic function of oligomeric a-syn and precedes overt neurodegeneration. Thus, it may constitute a readout for therapeutic approaches.

**Electronic supplementary material:**

The online version of this article (10.1007/s00429-017-1561-5) contains supplementary material, which is available to authorized users.

## Introduction

The common neuropathological hallmark of α-synucleinopathies, including Parkinson’s disease (PD) and dementia with Lewy bodies (DLB), is the deposition of aggregated α-synuclein (a-syn) within affected brain regions, paralleled by neuronal loss (Halliday et al. 2011). The putative function of a-syn has been implicated in regulation of the synaptic vesicle pool and neurotransmitter release due to its presynaptic localization in mature neurons and changes in synaptic transmission in a-syn knockdown and overexpression models (Iwai et al. 1995; Abeliovich et al. 2000; Murphy et al. 2000; Chandra et al. 2004; Nemani et al. 2010). The potential overlap of these functions of a-syn with its pathogenic effects in PD remains elusive (Lashuel et al. 2013). There is an increasing body of evidence showing a high neuronal toxicity of the oligomeric conformation of a-syn, whereas the derived aggregated, fibrillar conformation has been considered less detrimental. Oligomeric a-syn is elevated in the cerebrospinal fluid of PD patients (Tokuda et al. 2010) and its presence precedes neurodegeneration in brains of affected patients (Roberts et al. 2015). In vitro, a-syn oligomers induce toxicity in dopaminergic neuroblastoma cells in a time- and concentration-dependent manner (Danzer et al. 2007). Putative mechanisms of increased cell death include a pore-forming capacity of oligomeric a-syn (Conway et al. 2000; Reynolds et al. 2011). The artificial E57K mutant was previously shown to produce oligomer-related pathology in rat substantia nigra in vivo (Winner et al. 2011). A transgenic mouse model overexpressing high levels of E57K-mutant a-syn in neurons under control of the murine Thy1-promoter (Thy1-E57K) was recently established (Rockenstein et al. 2014). Compared to mice overexpressing human wild-type a-syn under the same promoter (Thy1-WTS) and to non-transgenic littermates (NTG), Thy1-E57K showed aggravated frontal and hippocampal pathology with regard to neuronal loss, reduction of the presynaptic marker synaptophysin, and context-dependent learning at the age of 8–10 months (Rockenstein et al. 2014). In contrast, the fibrillar conformation of a-syn has been considered less detrimental (Winner et al. 2011).

The integration of newborn neurons during adult neurogenesis is a useful model to study the effects of disease-related proteins on spine formation within the ageing brain (Mu et al. 2010). Adult newborn neuron integration was impaired in a previous WTS-transgenic mouse model (Winner et al. 2012). The impact of oligomeric a-syn species on adult newborn neurons is unknown to date. Therefore, in the current study, we characterize the integration deficit of newborn hippocampal neurons comparing the wild-type a-syn (Thy1-WTS) and oligomer-prone a-syn transgenic models (Thy1-E57K) to non-transgenic controls (NTG). We demonstrate neuritic pathology due to transgenic oligomeric a-syn species. This supports the hypothesis that oligomeric a-syn promotes synaptic dysfunction as an early event in PD pathogenesis.

## Materials and methods

### Animals

Animal experiments were conducted in accordance with the European Communities Council Directive of 24th November 1996 and were approved by the local governmental administrations for animal health (animal care use committee of the University of California, San Diego and “Regierung von Unterfranken”, Würzburg, Az. 55.2-2532.1-45/11). Generation of the WTS- and the E57K-transgenic mouse lines was described earlier (Rockenstein et al. 2002, 2014). WTS-transgenic mice overexpress human wild-type a-syn under the regulatory control of the mThy1 promoter (high-expressing line 61). E57K-transgenic mice overexpress human a-syn with an E57K point mutation under control of the same mThy1 promoter (high-expressing line 16). In all experiments, transgenic animals were compared to non-transgenic (NTG) wild-type littermate controls of the same C57BL6/DBA background (*n* = 6 per group).

### BrdU treatment and tissue processing

Animals (aged 3 months, *n* = 5 per genotype) received daily i.p. injections of 5-bromo-2-deoxyuridine (BrdU, 50 mg/kg) for 5 days and were sacrificed after 31 days. Euthanasia with xylazine/ketamine i.p. was followed by transcardial perfusion of animals with PBS followed by 4% paraformaldehyde for tissue fixation. Brains were dissected, postfixed for 6 h in 4% paraformaldehyde, and stored in 30% sucrose in 0.1 M phosphate buffer at 4 °C. 40 µm-thick brain sections were obtained on a sliding microtome and were stored in cryoprotectant solution (25% ethylene glycol, 25% glycerol in 0.1 M phosphate buffer) at − 20 °C. As BrdU labeling studies were conducted separately for Thy1-WTS and Thy1-E57K, NTG littermate controls were included in each experiment.

### Retrovirus-mediated labeling and analysis of newborn neurons

A Moloney murine leukemia retrovirus-based CAG–GFP plasmid was used as described earlier (Zhao et al. 2006). CAG–GFP drives the expression of enhanced green fluorescent protein (GFP) by the compound promoter CAG. A concentrated viral solution was titrated to 4 × 10^8^ pfu/ml. Mice were anaesthetized using a weight-adjusted i.p. dose of xylazine/ketamine and a stereotaxic frame (Kopf Instruments) was used for sequential bilateral infusion into the dentate gyrus (AP − 2.00 mm, ML ± 1.6 mm from bregma, DV − 2.3 mm from skull) of transgenic mice (WTS and E57K) and respective controls (*n* = 6 per group). A total volume of 1 µl was slowly infused (0.2 µl/min) followed by wound closure and a survival period of 31 days.

### Immunohistochemistry

The following primary antibodies were used: rt-anti-α-synuclein (15G7, Enzo Life Science, Germany, 1:50), rb-anti-Sox2 (Cell Signaling, 1:250), gt-anti-DCX (C18, Santa Cruz, 1:500), ms-anti-NeuN (Millipore, 1:200), ms-anti-PCNA (Santa Cruz, 1:50), rt-anti-BrdU (AbD Serotec, 1:500), ch-anti-GFP (Abcam, 1:500), and rb-anti-cleaved-caspase-3 (Cell Signaling, 1:1000). Secondary antibodies included donkey anti-mouse- and donkey anti-goat-biotinylated (1:1000, Jackson Immuno Research) for immunohistochemistry and respective secondary antibodies marked with fluorophores Alexa-488/568/Cy5 (Invitrogen, 1:500) for immunofluorescence. Immunofluorescence was conducted as described previously (Winner et al. 2012). Sections were blocked in 3% donkey serum/0.1% TritonX100 in TBS and incubated overnight with primary antibodies at 4 °C. Sections were incubated with secondary antibodies 3 h and washed again in TBS. Nuclei were counterstained with DAPI (ThermoFisher, final concentration 1:2000) and mounted on object glasses (Superfrost Slides, Menzel). The following DNA denaturation steps preceded anti-BrdU and anti-PCNA antibody incubation: 30 min in 2 M HCl at 37 °C, 10 min rinse in 0.1 M boric acid, pH 8.5. For immunohistochemical staining, sections were incubated in avidin–biotin–peroxidase solution (ABC-kit, Vector Laboratories, 1:100) for 1 h, followed by washing and incubation in 3.3-diaminobenzidine (DAB) for 5–10 min.

### Microscopy

All counting procedures were performed on blind-coded slides. Recordings were performed on a fluorescence microscope (Observer.Z1, Zeiss) and on a confocal laser scanning microscope (LSM710, Zeiss) using the ZEN black software. For dendrite growth analyses, on average, four GFP-positive newborn neurons in the dentate gyrus of each animal were imaged resulting in a cell number of 24 per group. For each neuron, *z*-series of antibody-enhanced GFP-signal at 1.5 µm were acquired spanning the whole extent of the neuron within the section. Maximum intensity projections were then analyzed with ImageJ and NeuronJ. Spine recordings were performed on unstained mounted sections to preserve signal intensity. We chose dendritic segments in the molecular layer, but not in the granule cell layer (GCL) for spine imaging. The estimated surface area of each spine was calculated as 0.785 × *D*_major_ × *D*_minor_, with *D*_major_ as the biggest diameter and *D*_minor_ as the smallest diameter of the respective spine. Mushroom spines were defined by their average estimated surface area from three measurements of at least 0.4 µm^2^ (Zhao et al. 2014).

For cell number and volume quantifications, every sixth section of the hippocampus was analyzed and values were multiplied by 6. For the differentiation analysis, 50 BrdU-positive cells were analyzed in the dentate gyrus of each animal; cells were randomly selected and analyzed by moving through the *z*-axis of each cell to exclude false-positive double labeling. Total numbers of newborn neurons were determined by multiplication of the total number of BrdU-positive cells by the ratio of BrdU/NeuN-positive cells. For the quantification of cell death, activated Caspase3 (aCaspase3) positive cells were counted in the granule cell layer and CA3 region of every 12th section. NeuN^+^ cells of the granule cell layer and the CA3 region were quantified within a randomly placed, 150 µm-wide counting frame in every 12th section and total cell numbers were estimated based on the ratio of the total area.

### Western blot analysis

For western blot analysis, tissue lysates were loaded onto 4–12% SDS/PAGE gels as described before (Rockenstein et al. 2014) and blotted onto polyvinylidene difluoride membranes, and incubated with rabbit polyclonal anti-a-syn antibody (1:1000, Millipore, AB5038), followed by horseradish peroxidase-tagged secondary antibodies (1:5000; Santa Cruz Biotechnology). Bands were visualized by enhanced chemiluminescence (PerkinElmer) and analyzed with a quantitative Versadoc XL imaging apparatus (Bio-Rad). Beta-actin (b-Actin) detected by anti-b-Actin antibody (1:3000) was the loading control.

### Statistical analysis

For statistical analysis with Prism (GraphPad Software), the significance level was set at *P* < 0.05. All parameters were compared using the two-sided student’s *t* test (regarding cell count, volume, dendrite length, number of branching points, spine density, and mushroom spine density) or a one-way ANOVA followed by a Tukey’s multiple comparison post hoc test (for the comparison of PDGF-WTS, Thy1-WTS, and Thy1-E57K as well as for the comparison of neuron numbers of NTG, Thy1-WTS, and Thy1-E57K).

## Results

### Impaired post-synaptic integration of newborn neurons in Thy1-WTS transgenic animals

**Fig. 1:**
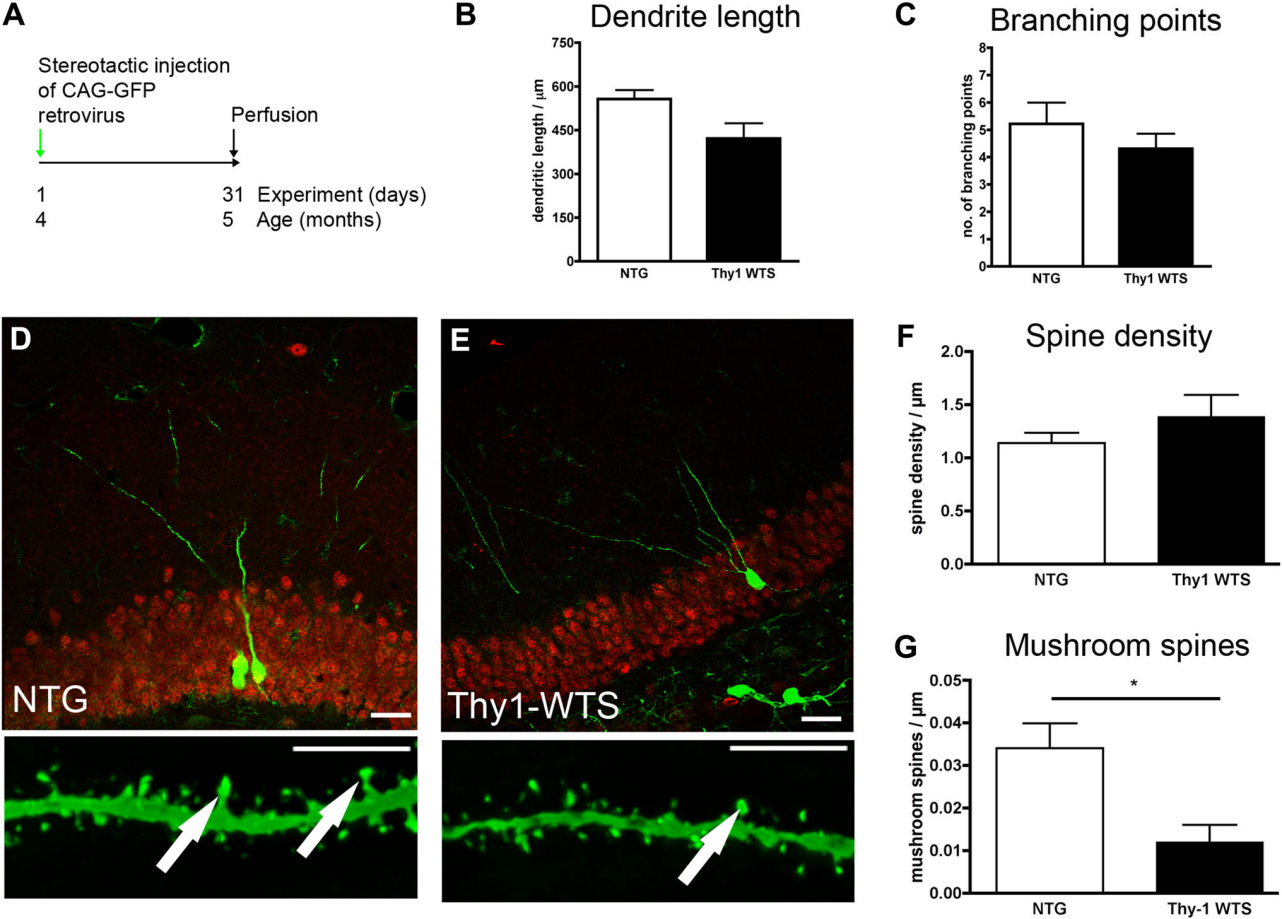
Impaired mushroom spine density in Thy1-WTS mice. **a** Experimental paradigm: CAG–GFP retrovirus was delivered to the hippocampus of 4-month-old animals and analysis was performed 1 month later. **b** Dendrite length was unchanged between NTG and Thy1-WTS mice. **c** Number of branching points was unchanged between NTG and Thy1-WTS mice. **d**, **e** Representative micrographs of GFP-labeled dendrites (upper line, scale bar 25 µm) and spines (lower line, scale bar 10 µm; arrows indicate mushroom spines) in NTG and Thy1-WTS mice. **f** Density of all spines was unchanged between NTG and Thy1-WTS. **g** Density of mushroom spines was significantly reduced in Thy1-WTS; *P < 0.05

We first analyzed the morphology of newborn hippocampal neurons in Thy1-WTS by retroviral labeling of dividing cells with GFP. Analysis was performed 1 month postinjection (Fig. 1a). Total dendritic length and the number of branching points of newborn neurons were not significantly changed when compared to NTG (see Table 1 for detailed results; Fig. 1b, c). The overall density of spines was unchanged when compared to NTG (Fig. 1f), but there was a significant reduction of mushroom spines (Fig. 1g)—a feature that has been shown before in PDGF-WTS and that indicates impaired spine maturation and postsynaptic integration of adult newborn neurons (Winner et al. 2012).

**Table 1:** Analysis of neurite morphology of adult newborn neurons in human wild-type a-syn transgenic animals (Thy1-WTS), human E57K-mutant a-syn transgenic animals (Thy1-E57K), and respective non-transgenic controls (NTG)

	NTG_(Thy1-WTS)_	Thy1-WTS	*P*	NTG_(Thy1-E57K)_	Thy1-E57K	*P*
*n*	6	6		6	6	
Dendritic length (µm)	557 ± 69.0	422 ± 127	0.06	337 ± 112	355 ± 118	0.68
Branching points (per cell)	5.20 ± 1.8	4.31 ± 1.4	0.36	3.96 ± 1.46	3.78 ± 1.48	0.75
Spine density (per µm)	1.14 ± 0.21	1.39 ± 0.20	0.08	0.94 ± 0.34	0.51 ± 0.12	0.0006
Density of mushroom spines (per µm)	0.034 ± 0.013	0.012 ± 0.007	0.038	0.127 ± 0.051	0.0635 ± 0.0251	0.0031

Numbers are given as mean ± SD and *P* values when compared to respective NTG

### Oligomer-prone E57K a-syn exacerbates integration deficit of newborn neurons

**Fig. 2:**
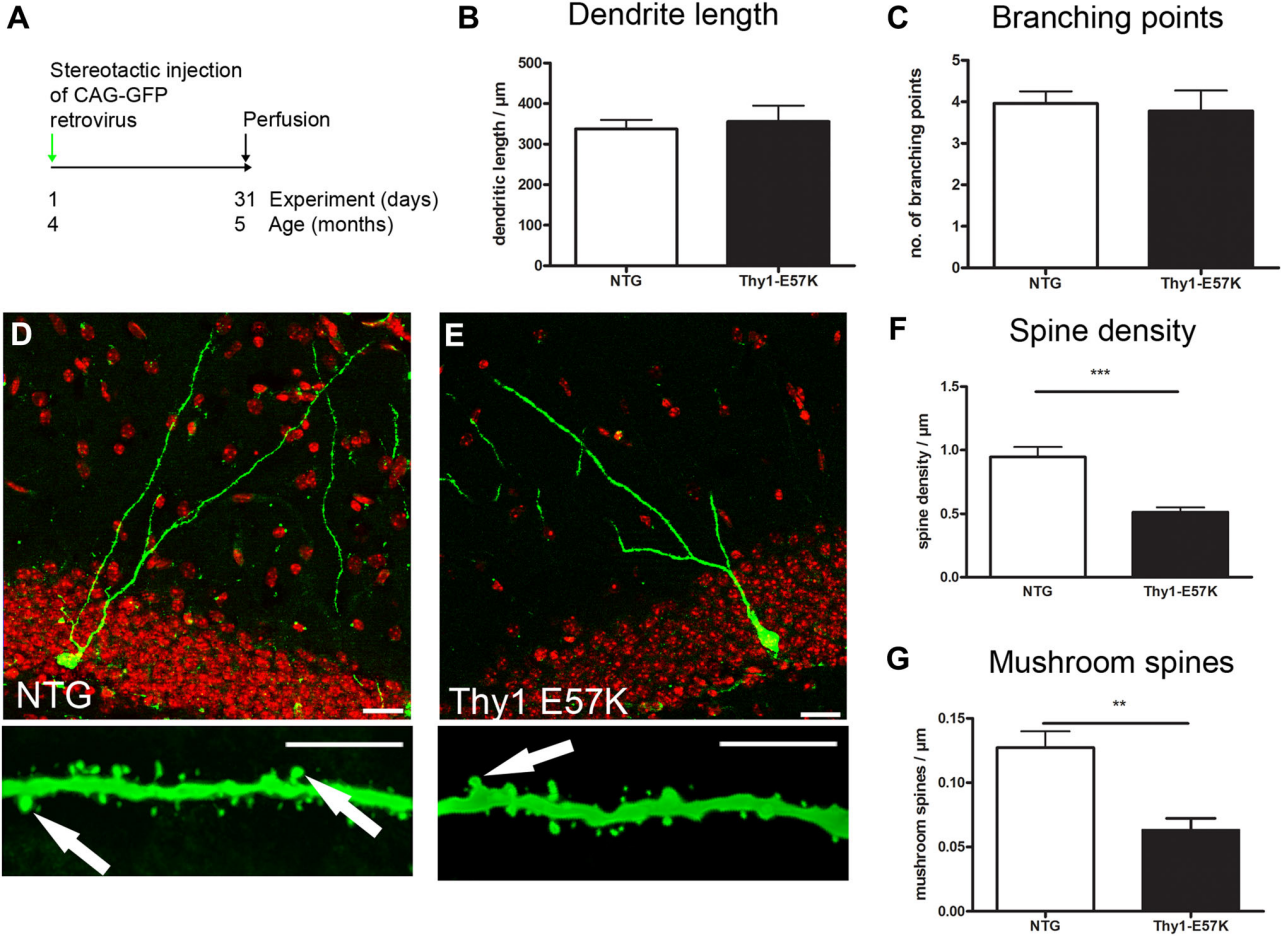
Impaired overall spine density in Thy1-E57K mice. **a** Experimental paradigm: CAG–GFP retrovirus was delivered to the hippocampus of 4-month-old animals; analysis was performed 1 month later. **b** Dendrite length was unchanged between NTG and Thy1-E57K mice. **c** Number of branching points was unchanged between NTG and Thy1-E57K mice. **d**, **e** Representative micrographs of GFP-labeled dendrites (upper line, scale bar 25 µm) and spines (lower line, scale bar 10 µm; arrows indicate mushroom spines) in NTG and Thy1-E57K mice. **f** Density of all spines was significantly reduced in Thy1-E57K. **g** Density of mushroom spines was significantly reduced in Thy1-E57K; **P < 0.01, ***P < 0.001

We previously showed a reduction of synaptic markers in the hippocampus of Thy1-E57K mice (Rockenstein et al. 2014) when compared to NTG and to Thy1-WTS. We thus analyzed the morphology of newborn neurons in Thy1-E57K by retroviral labeling (see Table 1 for detailed results; Fig. 2a). Similar to Thy1-WTS, we observed no outgrowth deficit regarding dendrite length and number of branching points (Fig. 2b, c). However, the overall density of spines was significantly reduced in Thy1-E57K (Fig. 2f). In addition, there was a significant reduction in mushroom spines (Fig. 2g), to a greater extent than what was observed in Thy1-WTS. In summary, the density of dendritic mushroom spines was reduced both in Thy1-WTS and in Thy1-E57K, whereas E57K a-syn had an additional strong negative effect on the density of all dendritic spines.

Cell-extrinsic a-syn in the molecular layer impairs mushroom spine density of newborn neurons in PDGF-WTS (Winner et al. 2012). We, therefore, analyzed the spatial relation of a-syn in the dendritic compartment of newborn neurons of Thy1-WTS and of Thy1-E57K. E57K a-syn was found directly adjacent to GFP-labeled newborn neurons including dendritic shaft, thin spines, and mushroom spines (SFig. 1). Within the dendrites and spines of newborn neurons, however, transgenic a-syn signal was at low levels in both Thy1-WTS and Thy1-E57K, consistent with the use of the same promoter. These data suggest that transgenic a-syn—as specifically labeled by the human-specific 15G7 a-syn antibody—within the axon terminals of the perforant path may impair mushroom spine density.

### Promoter-dependent influence of transgenic a-syn on adult neurogenesis

**Fig. 3:**
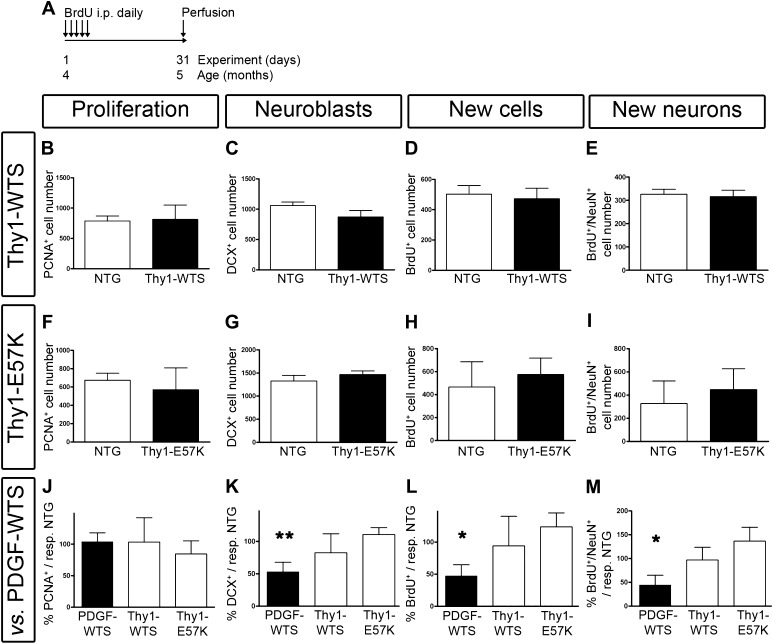
Differential effects of transgene promoters on adult hippocampal neurogenesis in a-syn transgenic mice. **a** Experimental paradigm was equivalent in all groups including BrdU-injections on 5 consecutive days and perfusion after 31 days. **b**–**e** Analysis of adult neurogenesis in Thy1-WTS showing unaffected numbers of PCNA-positive cells (**b**), DCX-positive cells (**c**), BrdU-positive cells (**d**), and BrdU/NeuN double-positive cells (**e**). **f**–**i** Analysis of adult neurogenesis in Thy1-E57K showing unaffected numbers of PCNA-positive cells (**f**), DCX-positive cells (**g**), BrdU-positive cells (**h**), and BrdU/NeuN double-positive cells (**i**). **j**–**m** Results from **b**–**i** were statistically compared to findings in the transgenic PDGF-WTS model that have been published previously (Winner et al. 2004). Shown are relative changes of adult neurogenesis. For all groups, the respective NTG values were set at 100%. All three mouse models showed no changes of PCNA-positive cells (**j**). When compared to Thy1-WTS and Thy1-E57K and normalized to respective NTG, significant reductions are found in PDGF-WTS for the numbers of DCX-positive cells (**k**), BrdU-positive cells (**l**), and BrdU/NeuN double-positive cells (**m**); *P < 0.05, **P < 0.01

In light of the spine alterations in Thy1-WTS and Thy1-E57K, we next analyzed proliferation and survival of newborn cells. In Thy1-WTS, the number of proliferating PCNA-positive cells in the subgranular zone of the hippocampal dentate gyrus was unchanged when compared to NTG (see Table 2 for detailed results; Fig. 3b). Likewise, the numbers of DCX-positive neuroblasts were unchanged in Thy1-WTS (Fig. 3c). We performed BrdU-labeling of newborn neurons at the age of 4 months and analyzed survival after 1 month. Thy1-WTS did not show differences in total numbers of BrdU-positive cells (Fig. 3d). There was no change in the ratio of neuronal differentiation (Table 2). The calculated numbers of BrdU-/NeuN-positive newborn neurons were also unchanged in Thy1-WTS (Fig. 3e). In summary, proliferation and survival of adult hippocampal newborn neurons were unchanged in the Thy1-WTS transgenic mouse model.

**Table 2:** Analysis of adult hippocampal neurogenesis in human wild-type a-syn transgenic animals (Thy1-WTS), human E57K-mutant a-syn transgenic animals (Thy1-E57K), and respective non-transgenic controls (NTG)

	NTG_(Thy1-WTS)_	Thy1-WTS	*P*	NTG_(Thy1-E57K)_	Thy1-E57K	*P*
*n*	5	5		5	5	
PCNA^+^ cells	788 ± 202	814 ± 236	0.84	674 ± 190	570 ± 239	0.42
DCX^+^ cells	1059 ± 136	873 ± 231	0.16	1325 ± 295	1465 ± 200	0.36
early DCX^+^	605 ± 68	504 ± 117	0.10	707 ± 187	763 ± 194	0.63
intermediate DCX^+^	245 ± 63	239 ± 78	0.89	425 ± 161	494 ± 57	0.34
late DCX^+^	210 ± 46	130 ± 58	**0.03**	194 ± 94	208 ± 48	0.74
BrdU^+^ cells	502 ± 142	472 ± 171	0.75	466 ± 220	576 ± 143	0.33
% NeuN^+^/BrdU^+^	65.0 ± 10.5	67.0 ± 14.2	0.79	71.0 ± 15.1	72.0 ± 17.8	0.92
BrdU^+^/NeuN^+^ cells	326 ± 53	316 ± 67	0.78	328 ± 194	448 ± 180	0.29

Numbers are given as mean ± SD and *P* values when compared to respective NTG. *P* value in bold indicates statistically significant difference

Oligomer-prone E57K a-syn previously showed enhanced neuronal toxicity when compared to WTS (Winner et al. 2011). In addition, the overall number of hippocampal NeuN-positive neurons was reduced in Thy1-E57K mice (Rockenstein et al. 2014). We thus analyzed adult newborn neuron proliferation and survival in the Thy1-E57K model. We found no differences when compared to NTG. In detail, proliferation was unchanged (see Table 2 for detailed results; Fig. 3f), the number of DCX-positive neuroblasts was unchanged (Fig. 3g), the total number of BrdU-positive cells was unchanged (Fig. 3h), the ratio of neuronal differentiation was unchanged (Table 2), and the calculated total number of newborn neurons was unchanged (Fig. 3i). For an analysis of young adult neuroblasts, i.e., newborn cells during their first 2 weeks of neuronal maturation, we analyzed the different morphological subtypes of DCX-positive cells (SFig. 2a–d). We observed a reduction of the number of late-stage neuroblasts in Thy1-WTS, but there was no difference of young and intermediate neuroblasts in Thy1-WTS and in Thy1-E57K (Table 2, SFig. 2). Taken together, adult neurogenesis is neither affected by WTS nor by E57K in the Thy1-transgenic model.

Since a significant loss of neurons was reported in the CA3 region of 8–10 months old Thy1-WTS and Thy-E57K mice (Rockenstein et al. 2014), we analyzed neuronal loss at the age of 4 months. Quantifying activated Caspase3 (aCaspase3)-positive cells, the total number of neurons, and the volume for both the granule cell layer and the CA3 region, we found no significant differences in a-syn transgenic mice (STable 1, SFig. 3). This indicates that in the adult hippocampus of Thy1-WTS and Thy1-E57K mice, apoptosis-mediated neurodegeneration occurs after 4 months of age.

In light of these observations of unaffected hippocampal neurogenesis in Thy1-promoter-based a-syn models, we statistically compared the current findings to previously published quantifications of adult neurogenesis in PDGF-WTS by Winner et al. (2004) which were conducted using the same paradigm. When normalized to respective NTG, there is a significant promoter dependence of the effects of transgenic a-syn on the numbers of DCX-positive neuroblasts (Fig. 3k), BrdU-positive cells (Fig. 3l), and newborn neurons (Fig. 3m).

In summary, whereas the PDGF-WTS model shows a pronounced defect of hippocampal proliferation and neurogenesis at 4 months, these parameters remain unchanged in the Thy1-WTS and the Thy1-E57K models, but there is an integration phenotype.

### Late transgenic expression of WTS and oligomer-prone E57K a-syn in the Thy1-model

**Fig. 4:**
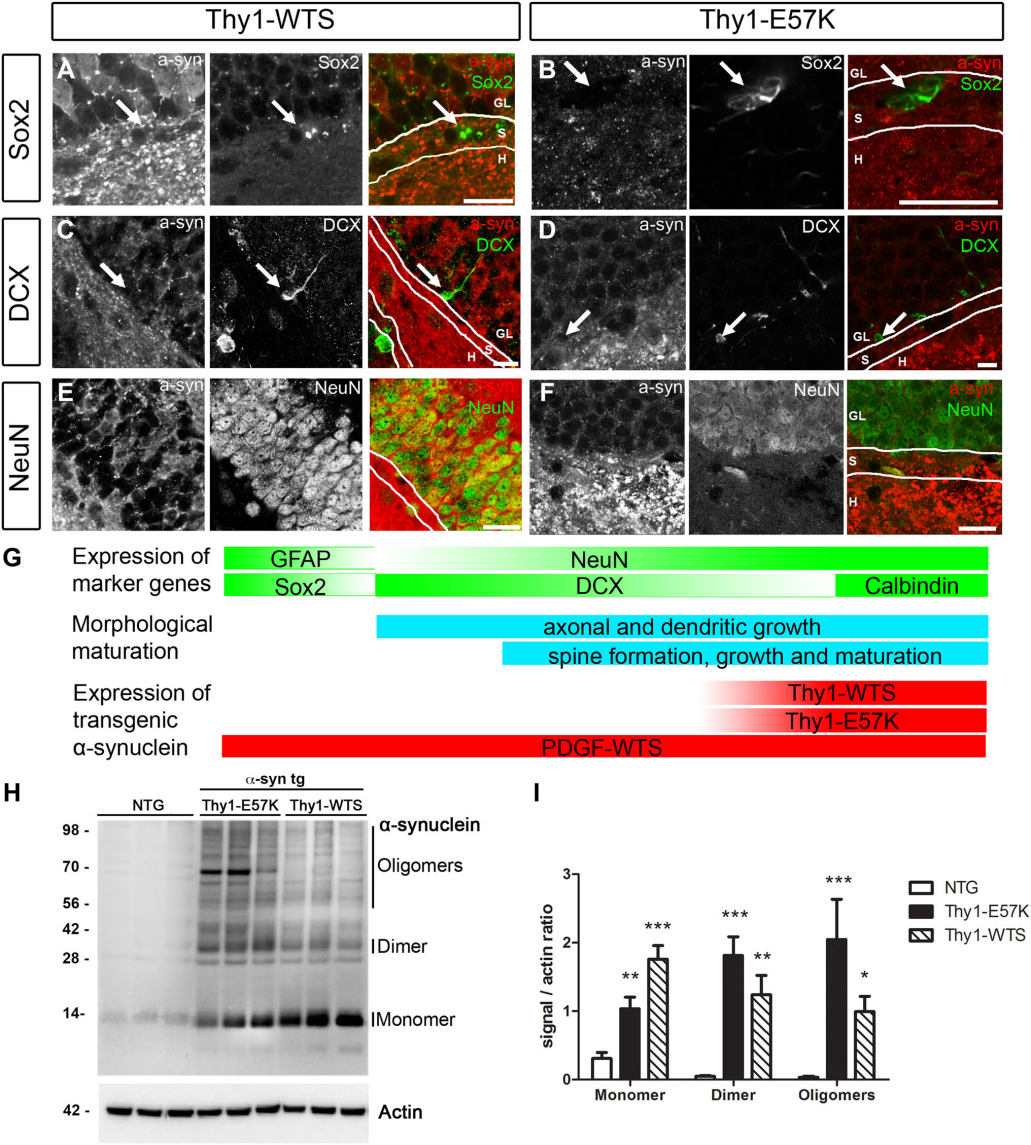
Low intrinsic, but high extrinsic transgene expression in adult neuroblasts of Thy1-WTS and Thy1-E57K mice. **a**–**f** Colocalization analysis of transgenic a-syn at different stages of adult newborn neuron development. **a**, **b** Sox2-positive stem cells (arrows) were negative for transgenic a-syn in Thy1-WTS and Thy1-E57K. **c**, **d** DCX-positive hippocampal neuroblasts (arrows) were only partly co-labeled with a-syn antibody in Thy1-WTS and Thy1-E57K. **e**, **f** Expression of transgenic a-syn in the dentate gyrus was mainly confined to mature, NeuN-positive granule cells. High expression was noted in the hilus and in the molecular layer. *GL* granule cell layer, *S* subgranular zone, *H* hilus. **g** Model of the temporal expression pattern of transgenic a-syn under the control of the PDGF- and Thy1-promoters. **h** Representative western blot and **i** analysis of the levels of a-syn in the hippocampus, showing that highest expression levels of monomeric a-syn (14 kDa) are found in Thy1-WTS, whereas dimers (28 kDa) and higher molecular weight oligomers (> 42 kDa) are predominantly present in Thy1-E57K. Scale bars 25 µm

We next addressed how the temporal and spatial expression patterns of the Thy1-promoter and the PDGF-promoter were different. In the hippocampus of adult PDGF-WTS, we have previously shown transgene expression in Sox2-positive neural stem cells, DCX-positive neuroblasts and NeuN-positive neurons (Winner et al. 2012). In Thy1-WTS, a-syn was detected neither in Sox2-positive adult hippocampal stem cells (Fig. 4a) nor in DCX-positive neuroblasts (Fig. 4c). Expression was detected in NeuN-positive cells along with strong protein expression in the hilus and in the molecular layer (Fig. 4e). Similarly, in Thy1-E57K, transgenic a-syn was not expressed in Sox2-positive stem cells (Fig. 4b) and DCX-positive neuroblasts (Fig. 4d). Expression in the somal compartment of dentate granule cells was weak, whereas highest expression was found in the molecular layer and the hilus (Fig. 4f). In conclusion, other than in PDGF-WTS, where transgenic a-syn is present at all stages of newborn neuron development, in Thy1-WTS and Thy1-E57K, the transgene is not present at the stem cell and neuroblast stages, but has a strong overall expression in the adult hippocampus (Fig. 4g).

We additionally confirmed the presence of transgenic a-syn by Western blot of the dissected hippocampus of NTG, Thy1-E57K, and Thy1-WTS (Fig. 4h). As expected, the total amount of a-syn was increased in Thy1-WTS and Thy1-E57K when compared to NTG (Fig. 4i). Abundant a-syn oligomers were present in Thy1-E57K, whereas Thy1-WTS showed a significant increase in monomeric a-syn when compared to NTG.

## Discussion

In the current work, we compared the effect of wild-type and oligomerizing a-syn on adult hippocampal neurogenesis. To this end, we analyzed adult hippocampal neurogenesis in two transgenic mouse models of α-synucleinopathy, overexpressing WTS and E57K-mutant a-syn under control of the Thy1 promoter. We found that in 1-month-old newborn neurons, transgenic WTS reduced mushroom spine density and transgenic E57K-mutant a-syn additionally reduced the density of all spines. In both transgenic groups, adult neurogenesis was unaffected in terms of numbers of proliferating and surviving cells, and we observed no overt neurodegeneration in the dentate gyrus. Furthermore, comparison with PDGF-WTS neurogenesis data shows that the effect of transgenic a-syn on adult cellular plasticity is promoter-dependent and may be related to absence of Thy1-regulated a-syn expression in neural stem/progenitor cells and neuroblasts. These data suggest that the effect of a-syn on adult neurogenesis depends on cell-autonomous expression in neural stem/progenitor cells and that reduction of post-synaptic spine density may constitute an early pathogenic function of oligomeric a-syn.

### Mushroom spine loss of newborn neurons as a common phenotype of a-syn transgenic mice

In the current study, mushroom spine density of newborn neurons was significantly reduced in Thy1-WTS and Thy1-E57K, similar to our previous observation in PDGF-WTS (Winner et al. 2012). Mushroom spines have been suggested to mediate particularly strong and stable synaptic input, based on their large head size, the enrichment in F-actin, and their relatively low motility (Sala 2002; Kasai et al. 2003). A-syn, in turn, impairs microtubule-dependent cytoskeleton changes (Prots et al. 2013). As overall neurite morphology is rather fixed in the late-stage newborn neuron development, late expression of Thy1-regulated a-syn may thus specifically impair dendritic spines, whereas dendrite length and dendritic branching remain unaffected. In light of the reduction of overall hippocampal synaptophysin at 8–10 months (Rockenstein et al. 2014), we suppose that the mushroom spine reduction persists or aggravates at later time points after cell birth. However, we cannot exclude a slowdown of mushroom spine maturation with normalized densities at later time points, because spine motility is highest from 1 to 2 months after cell birth (Zhao et al. 2006). Interestingly, in a mouse model overexpressing A30P-mutant a-syn under control of the Thy1 promoter, spine formation on adult-born granule cells of the olfactory bulb was also compromised beginning 3–4 weeks after labeling which was related to the critical time point of spine formation (Neuner et al. 2014).

### Novel pathogenic effect of oligomer-prone a-syn on spine density of newborn neurons

Overall spine density of newborn neurons was intact in Thy1-WTS, but severely reduced in Thy1-E57K. As we analyzed two analogous transgenic mouse models, we conclude that oligomer-prone a-syn exacerbates spine pathology of newborn neurons. Reduction of overall synaptophysin in the hippocampus and loss of hippocampal NeuN-positive mature neurons is more severe in Thy1-E57K than in Thy1-WTS at the age of 8–10 months (Rockenstein et al. 2014). Our analogous analysis at 4 months revealed unchanged hippocampal neuron numbers, suggesting that a-syn oligomerization-mediated mushroom spine pathology of newborn neurons precedes overt neurodegeneration. This seems to be a time- and dose-dependent effect, since the spatial expression pattern of transgenic a-syn in the granule cell layer and the hilus was similar when comparing 4-month-old (Fig. 4) and 8–10-month-old (Rockenstein et al. 2014) animals.

In post-mortem tissue of DLB cases, high amounts of aggregated a-syn were found in the presynaptic compartment along with loss of postsynaptic dendritic spines, suggesting that presynaptic a-syn might be a trigger of functional impairment (Kramer and Schulz-Schaeffer 2007; Burke and O’Malley 2013). In line with this observation, transgenic a-syn did not substantially colocalize with newborn neurons’ dendrites in Thy1-WTS and Thy1-E57K (Rockenstein et al. 2014; SFig. 1). Transgenic a-syn may thus be mainly present within axon terminals of the perforant path. Indeed, axonal pathology and dysregulation of axonal transport proteins precede dopaminergic neuron loss in an AAV-model of synucleinopathy (Chung et al. 2009). Thus, spine loss of newborn neurons may represent an early feature of pathology in the Thy1-E57K model and might serve as a marker of disease progression.

A-syn spreads among neuronal circuits leading to the continuous propagation of pathology in α-synucleinopathies (Desplats et al. 2009; Hansen et al. 2011; Luk et al. 2012). Oligomeric a-syn is more disposed to propagation, which may explain increased pathology in Thy1-E57K (Peelaerts et al. 2015). Extracellular presence of oligomeric a-syn in acute hippocampal slices impaired long-term potentiation in the CA1 pyramidal synapse and increased basal synaptic transmission (Diógenes et al. 2012). Another study on hippocampal neurons showed that extracellular oligomeric a-syn amplifies glutamate-induced toxicity (Hüls et al. 2011). High levels of a-syn oligomers are present in the hippocampus of Thy1-E57K mice (Fig. 4) and may thus elicit similar excitotoxic effects.

Most dendritic spines of adult-born neurons integrate by competing for the existing synapses, indicating that spine-formation is activity-driven (Toni et al. 2007). Moreover, about 1 month after neuronal birth, long-term potentiation is facilitated by increased potentiation amplitude and decreased induction thresholds (Ge et al. 2007). Accordingly, spine formation may be compromised in Thy1-E57K mice due to a reduction of presynaptic signaling. Reduced neurotransmitter release has, indeed, been shown in a-syn models and loss of hippocampal synaptophysin in Thy1-E57K is an indirect sign of decreased synaptic input (Rockenstein et al. 2014). Neurotransmitter release was impaired upon a-syn overexpression in primary hippocampal and midbrain neurons and in hippocampal slices from a-syn transgenic mice (Nemani et al. 2010). Changes in vesicle release might be caused by a direct effect of a-syn on SNARE proteins and vesicle priming (Chandra et al. 2005; Larsen et al. 2006). Alternatively, we cannot exclude low levels of propagation of E57K a-syn into newborn neurons or cell-intrinsically expressed E57K a-syn which may have contributed to the observed spine loss in a cell-autonomous manner.

### Early expression of transgenic a-syn is necessary to impair adult neuronal survival

Our data of late transgene expression under regulation of the Thy1 promoter are compatible with reports, showing that promoter activity of Thy1 is absent during embryonic development, has an onset around birth, and reaches a plateau 1 month postnatally (Aigner et al. 1995; Caroni 1997; Lüthi et al. 1997; Wiessner et al. 1999; Kahle et al. 2001). The PDGFβ-promoter, on the other hand, is active already during the embryonic development (Sasahara et al. 1991, 1992). In the PDGF-WTS model, we have previously shown that PDGFβ-promoter-driven a-syn is also expressed in adult hippocampal stem cells and neuroblasts (Winner et al. 2012), contrasting to our current results in the Thy1-models. Therefore, our suggested expression kinetics (Fig. 4g) are based on direct expression data in neuroblasts together with the correlation to embryonic development. These results are corroborated by the analysis of two independently generated transgenic mouse models and by analogous results from the expression of transgenic a-syn in the subventricular zone and olfactory bulb of Thy1-WTS mice (Schreglmann et al. 2015). We observed a significant reduction of the late-stage neuroblasts in Thy1-WTS (SFig. 2h). However, due to unchanged dendrite length and unchanged BrdU-positive cell numbers in Thy1-WTS, a delay of maturation or a major loss of late-stage neuroblasts is unlikely. Given the discrepancy between Thy1- and PDGF-promoter-based mouse models in the adult neurogenic niche, we suggest that cell-autonomous overexpression of a-syn during the stem- and progenitor cell state is necessary to impair survival of their progeny.

Indeed, the matter of temporo-spatial promoter regulation is well known from many studies of adult neurogenesis in transgenic models of Alzheimer’s disease (Mu and Gage 2011). Different types of transgenic mouse models overexpressing human amyloid precursor protein (hAPP) showed a reduction of proliferation and newborn neuron survival in the adult hippocampus (Haughey et al. 2002; Crews et al. 2010). However, lack of cell-autonomous hAPP-expression within adult neural stem/progenitor cells spared adult neurogenesis (Yetman and Jankowsky 2013). Our data thus argue for a deleterious effect of WTS within immature adult neural progenitors rather than a developmental defect. For this reason, the PDGF-WTS mouse model provides a recapitulation of a-syn-induced stem cell pathology. However, for the matter of progressive spine loss followed by neurodegeneration, Thy1-based models seem to be of choice.

## Electronic supplementary material

Below is the link to the electronic supplementary material.

Supplementary material 1 Supplemental Fig. 1 Direct spatial relation of transgenic a-syn and newborn neuron dendrites and spines. Immunohistochemical localization of WTS (A) and E57K (B) a-syn in the molecular layer of transgenic mice 1 month after GFP-labeling of newborn neurons. In both groups, transgenic a-syn is found at high levels in the molecular layer and in the hilus, in direct proximity to the dendrites of GFP-positive newborn neurons, including thin spines and mushroom spines (labeled by intersecting bars). Scale bars overview 10 µm, insets 2 µm (TIFF 9011 kb)

Supplementary material 2 Supplemental Fig. 2 Subpopulations of neuroblasts in a-syn transgenic mice. (A) Low magnification overview of doublecortin (DCX) staining of the dentate gyrus. (B–D) Sample images of DCX-positive cells with early, intermediate, and late-stage morphology, respectively. (E–H) DCX-positive cells in Thy1-WTS animals. The total number of DCX-positive cells was unchanged (E; compare Fig. 3c). The subpopulations of early (F) and intermediate (G) neuroblasts were unchanged in Thy1-WTS. There was a significant reduction of late-stage neuroblasts when compared to NTG (H). (I–L) Quantification of DCX-positive cells in Thy1-E57K animals. Similar to Thy1-WTS, there were no significant changes in the total number of neuroblasts (I, compare Fig. 3g) as well as the subpopulations of early (J) and intermediate (K) neuroblasts. In Thy1-E57K, there was no change in the number of the late-stage neuroblasts (L). Scale bars (A) 200 µm, (B–D) 20 µm. * *P* < 0.05 (TIFF 1354 kb)

Supplementary material 3 Supplemental Fig. 3 Hippocampal cell death and number of neurons at 4 months of age. (A) Representative micrograph of aCaspase3^+^ cells in the granule cell layer. (B) Representative overview image of NeuN^+^ cells in the hippocampus. *GCL* granule cell layer. (C–H) Quantification of aCaspase3^+^ cells, NeuN^+^ cells, and volume in the granule cell layer and the CA3 region. No significant differences were observed between the groups. Scale bars (A) 20 µm, (B) 500 µm (TIFF 1370 kb)

Supplementary material 4 (DOCX 115 kb)
