# Supplementary figures and images for: Oligomer-prone E57K-mutant alpha-synuclein exacerbates integration deficit of adult hippocampal newborn neurons in transgenic mice
Source: Brain Struct Funct. 2017 Nov 9;223(3):1357–68. doi: 10.1007/s00429-017-1561-5 (PMC5869938; doi:10.1007/s00429-017-1561-5)

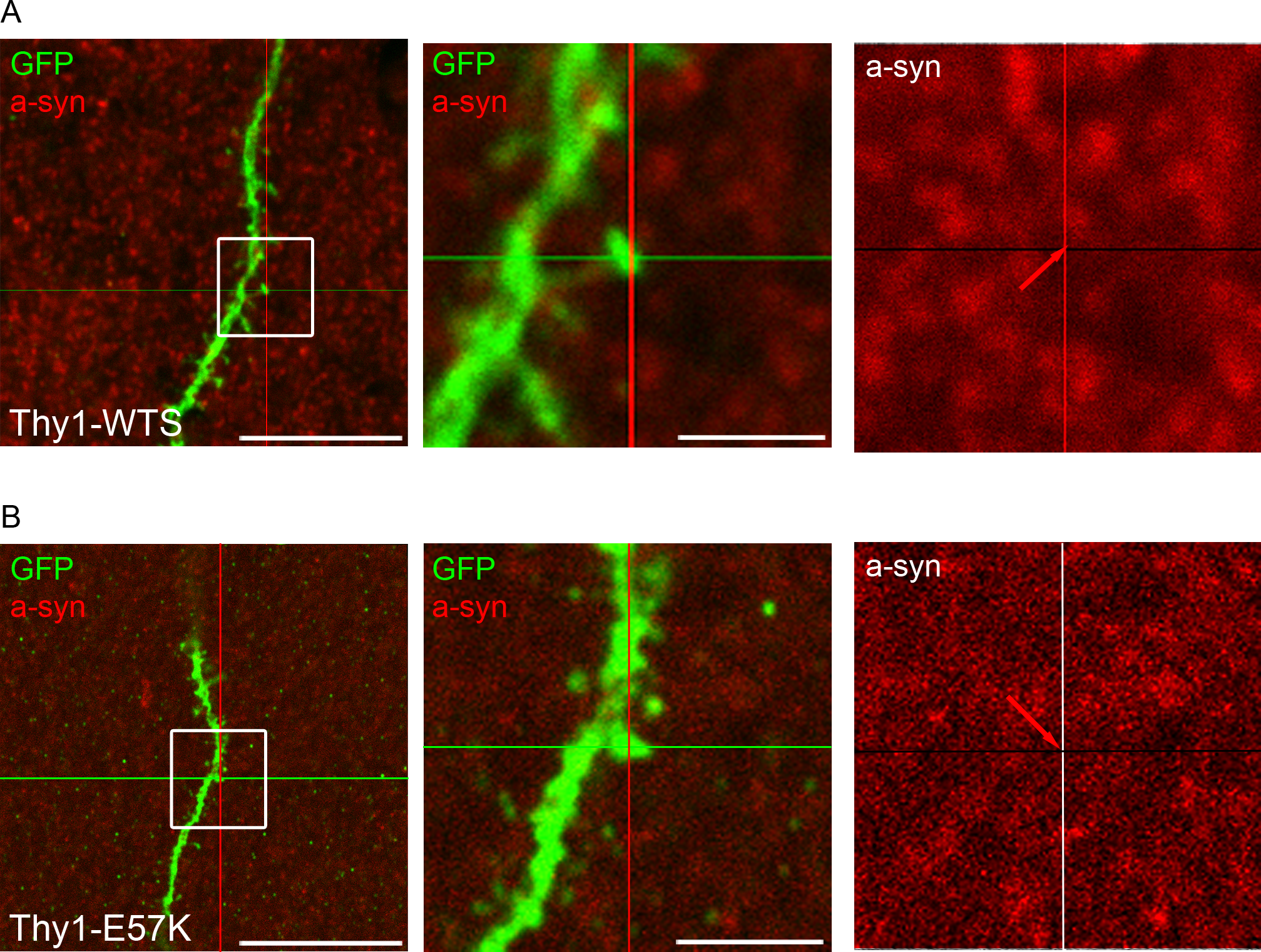

Supplement: Supplementary file 1 — Supplementary material 1 Supplemental Fig. 1 Direct spatial relation of transgenic a-syn and newborn neuron dendrites and spines. Immunohistochemical localization of WTS (A) and E57K (B) a-syn in the molecular layer of transgenic mice 1 month after GFP-labeling of newborn neurons. In both groups, transgenic a-syn is found at high levels in the molecular layer and in the hilus, in direct proximity to the dendrites of GFP-positive newborn neurons, including thin spines and mushroom spines (labeled by intersecting bars). Scale bars overview 10 µm, insets 2 µm (TIFF 9011 kb) [file 429_2017_1561_MOESM1_ESM.tif]

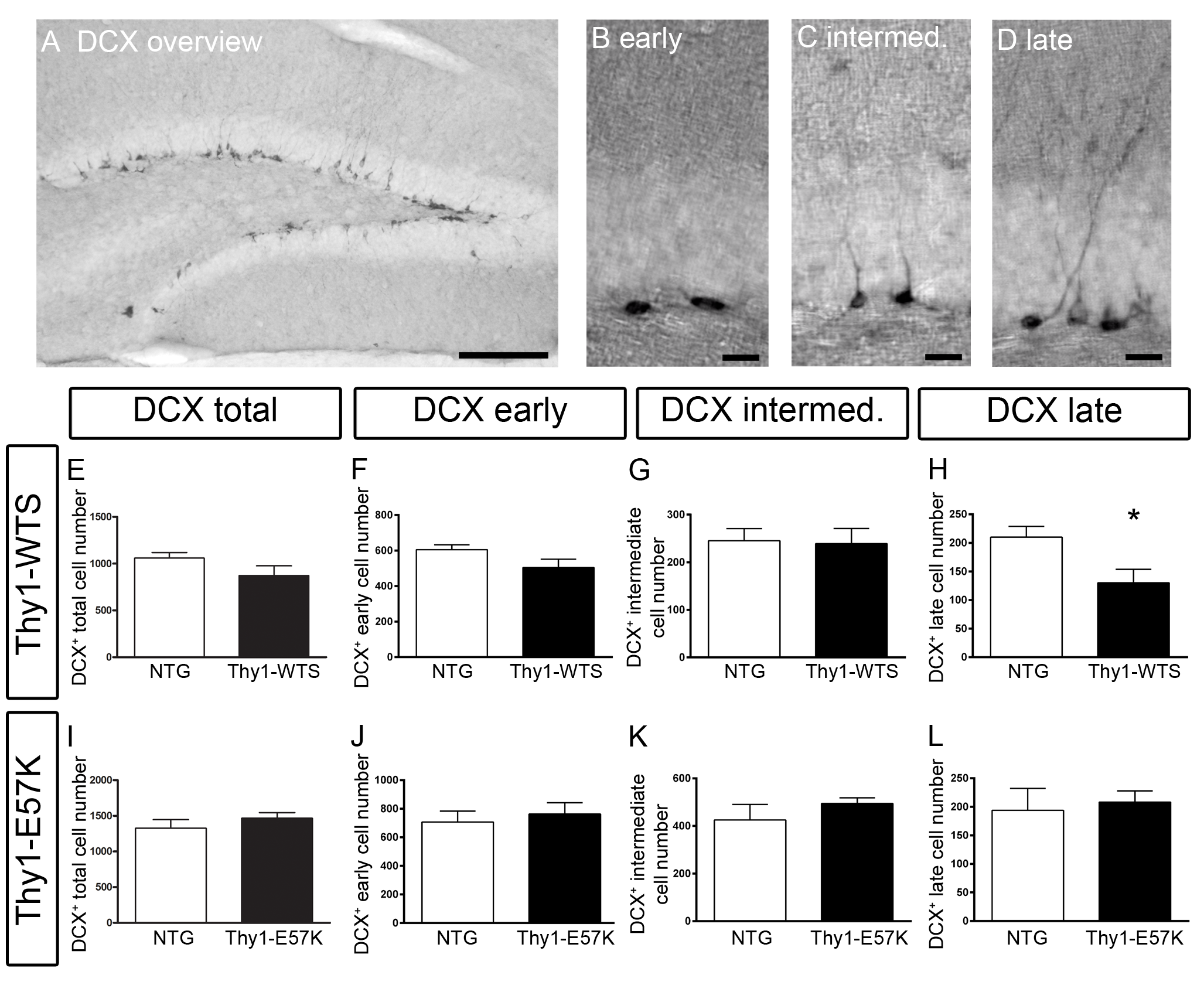

Supplement: Supplementary file 2 — Supplementary material 2 Supplemental Fig. 2 Subpopulations of neuroblasts in a-syn transgenic mice. (A) Low magnification overview of doublecortin (DCX) staining of the dentate gyrus. (B–D) Sample images of DCX-positive cells with early, intermediate, and late-stage morphology, respectively. (E–H) DCX-positive cells in Thy1-WTS animals. The total number of DCX-positive cells was unchanged (E; compare Fig. 3c). The subpopulations of early (F) and intermediate (G) neuroblasts were unchanged in Thy1-WTS. There was a significant reduction of late-stage neuroblasts when compared to NTG (H). (I–L) Quantification of DCX-positive cells in Thy1-E57K animals. Similar to Thy1-WTS, there were no significant changes in the total number of neuroblasts (I, compare Fig. 3g) as well as the subpopulations of early (J) and intermediate (K) neuroblasts. In Thy1-E57K, there was no change in the number of the late-stage neuroblasts (L). Scale bars (A) 200 µm, (B–D) 20 µm. * P < 0.05 (TIFF 1354 kb) [file 429_2017_1561_MOESM2_ESM.tif]

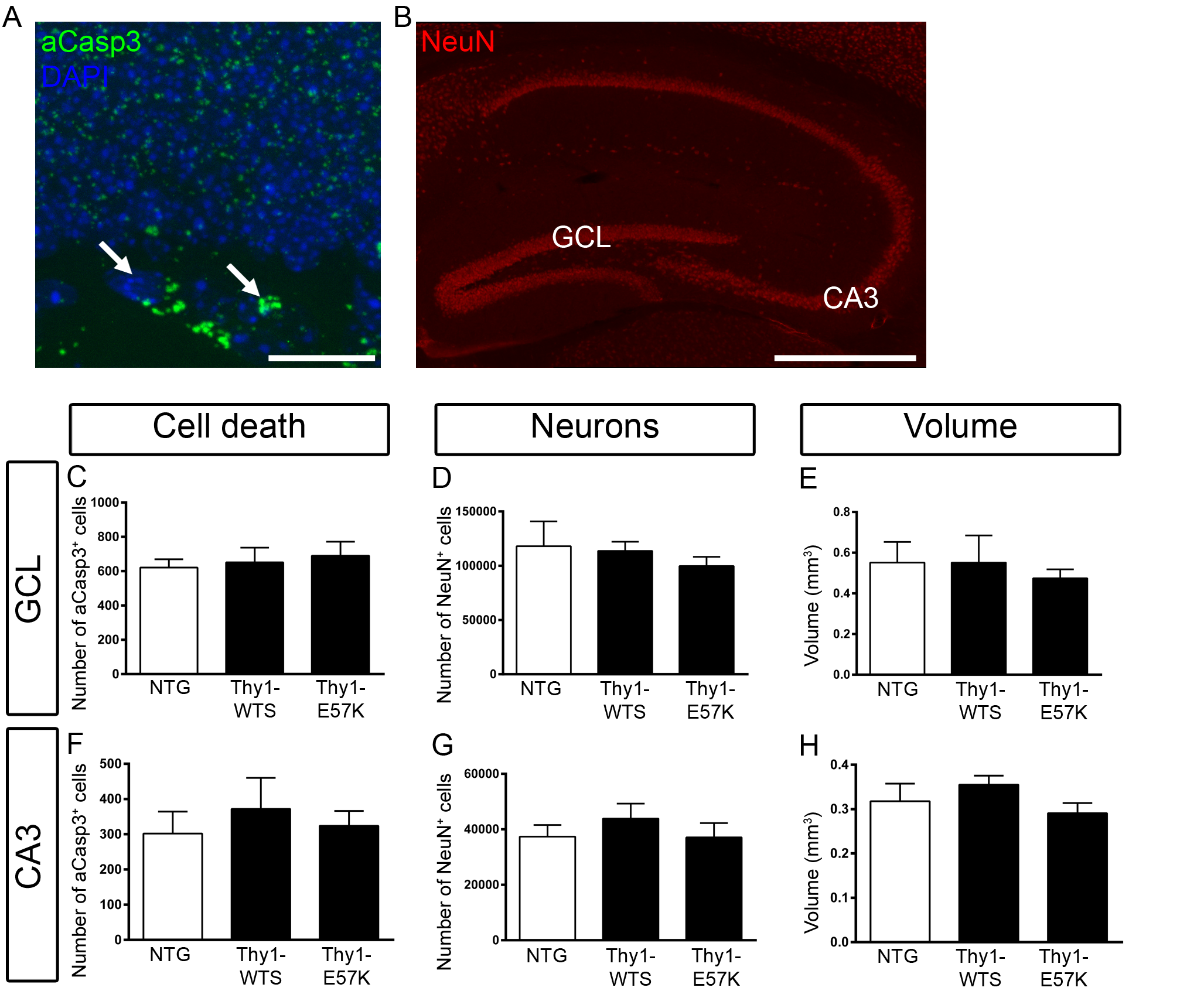

Supplement: Supplementary file 3 — Supplementary material 3 Supplemental Fig. 3 Hippocampal cell death and number of neurons at 4 months of age. (A) Representative micrograph of aCaspase3+ cells in the granule cell layer. (B) Representative overview image of NeuN+ cells in the hippocampus. GCL granule cell layer. (C–H) Quantification of aCaspase3+ cells, NeuN+ cells, and volume in the granule cell layer and the CA3 region. No significant differences were observed between the groups. Scale bars (A) 20 µm, (B) 500 µm (TIFF 1370 kb) [file 429_2017_1561_MOESM3_ESM.tif]
