# Supplementary material for: Oligomer-prone E57K-mutant alpha-synuclein exacerbates integration deficit of adult hippocampal newborn neurons in transgenic mice
Source: Brain Struct Funct. 2017 Nov 9;223(3):1357–68. doi: 10.1007/s00429-017-1561-5 (PMC5869938; doi:10.1007/s00429-017-1561-5)
Supplement: Supplementary file 4 — Supplementary material 4 (DOCX 115 kb) [file 429_2017_1561_MOESM4_ESM.docx]

Supplemental Table 1. **Numbers of neurons and apoptotic cells** in human wild-type a-syn transgenic animals (Thy1-WTS), human E57K-mutant a-syn transgenic animals (Thy1-E57K) and shared non-transgenic controls (NTG). Numbers are given as mean ± SD and *P*-values of one-way ANOVA. ^‡^ *P*<0.05 for Thy1-E57K vs. NTG (Tukey multiple comparison test).

|  | **NTG** | **Thy1-WTS** | **Thy1-E57K** | ***P*** |
| --- | --- | --- | --- | --- |
| n | 6 | 5 | 6 |  |
| aCaspase 3^+^ cells |  |  |  |  |
| Granule cell layer | 621 ± 120 | 651 ± 193 | 690 ± 202 | 0.79 |
| CA3 | 302 ± 153 | 372 ± 197 | 324 ± 103 | 0.75 |
| NeuN^+^ cells |  |  |  |  |
| Granule cell layer | 118,113 ± 55,770 | 113,689 ± 18,889 | 99,779 ± 20,757 | 0.68 |
| CA3 | 37,373 ± 10,306 | 43,858 ± 12,059 | 37,091 ± 12,530 | 0.58 |
| Volume |  |  |  |  |
| Granule cell layer (mm^3^) | 0.552 ± 0.248 | 0.552 ± 0.297 | 0.474 ± 0.108 | 0.80 |
| CA3 (mm^3^) | 0.318 ± 0.097 | 0.355 ± 0.045 | 0.291 ± 0.056 | 0.35 |
